# Common ELF1 deletion in prostate cancer bolsters oncogenic ETS function, inhibits senescence and promotes docetaxel resistance

**DOI:** 10.18632/genesandcancer.182

**Published:** 2018-05

**Authors:** Justin A. Budka, Mary W. Ferris, Matthew J. Capone, Peter C. Hollenhorst

**Affiliations:** ^1^ Medical Sciences, Indiana University School of Medicine, Bloomington, Indiana, USA

**Keywords:** ELF1, prostate cancer, tumor suppressor, ETS, chemotherapy resistance

## Abstract

ETS family transcription factors play major roles in prostate tumorigenesis with some acting as oncogenes and others as tumor suppressors. ETS factors can compete for binding at some cis-regulatory sequences, but display specific binding at others. Therefore, changes in expression of ETS family members during tumorigenesis can have complex, multimodal effects. Here we show that ELF1 was the most commonly down-regulated ETS factor in primary prostate tumors, and expression decreased further in metastatic disease. Genome-wide mapping in cell lines indicated that ELF1 has two distinct tumor suppressive roles mediated by distinct cis-regulatory sequences. First, ELF1 inhibited cell migration and epithelial-mesenchymal transition by interfering with oncogenic ETS functions at ETS/AP-1 cis-regulatory motifs. Second, ELF1 uniquely targeted and activated genes that promote senescence. Furthermore, knockdown of ELF1 increased docetaxel resistance, indicating that the genomic deletions found in metastatic prostate tumors may promote therapeutic resistance through loss of both RB1 and ELF1

## BACKGROUND

More than half of prostate tumors have a chromosomal rearrangement that results in aberrant expression of an ETS transcription factor that is not normally expressed in prostate cells. The most common of these rearrangements is the fusion of the ETS family member *ERG* to the promoter and 5′ UTR of the *TMPRSS2* gene, occurring in approximately 50% of prostate tumors. Other commonly rearranged ETS family members include *ETV1*, and *ETV4* [1, 2]. These ETS factors, when coupled with additional oncogenic mutations, drive prostate tumorigenesis [3–5]. However, there are many other ETS factors expressed in normal prostate epithelia, and some of these can act as tumor suppressors. The tumor suppressive mechanisms of these normally expressed ETS factors and their interplay with oncogenic ETS factors are not well understood.

Because ETS factors bind to similar DNA sequences [6], there is the possibility of binding site competition between oncogenic ETS factors and ETS factors expressed in normal prostate cells. There are approximately 14 members of the ETS family which are normally expressed within the prostate [7]. *EHF* and *SPDEF* are the two most highly expressed ETS factors in normal prostate, and both are reported as being down-regulated in prostate tumors, resulting in increased epithelial to mesenchymal transition (EMT), cell migration, and invasion [8–12]. These are similar phenotypes to those that occur when oncogenic ETS factors are expressed in prostate epithelial cells [13–15], and chromatin immunoprecipitation analysis indicates that EHF can compete with ERG for occupancy of the *EZH2* and *NKX3.1* promoters [16]. Inactivating point mutations and deletions of the ETS factor *ERF* were recently identified in about 4% of prostate tumors, and these mutations are able to recapitulate phenotypes of ERG overexpression; furthermore, ChIP-seq analyses indicate that ERF and ERG compete for binding throughout the genome [17]. Additional ETS factors have been shown to have tumor suppressive functions in the prostate. The interstitial deletion that most commonly results in the TMPRSS2/ERG fusion deletes one copy of the ETS gene *ETS2.* The loss of *ETS2* from this deletion, is associated with poor patient outcomes and promotes prostate cancer progression in a mouse model [18].

Evidence from Ewing's sarcoma further supports the idea that binding site competition between ETS factors could contribute to tumorigenesis. In this type of sarcoma, 85% of patients have a chromosomal translocation that results in the fusion of the *EWSR1* gene with the ETS factor *FLI1* [19]. We have recently reported that EWSR1-FLI1 and the oncogenic ETS expressed in prostate cancer bind to similar cis-regulatory sequences and activate transcription through overlapping mechanisms [20]. It has been reported that transcriptional repression mediated by EWS-FLI1 can occur due to binding site competition and displacement of the endogenously expressed ETS protein ELF1 [21]. ELF1 is also normally expressed within the prostate, but its function in this tissue has not been characterized.

*ELF1* is a ubiquitously expressed ETS gene. Previous studies on ELF1's function in cancer indicate both oncogenic and tumor suppressive roles. Studies in endometrial carcinoma, epithelial ovarian carcinoma, and non-small cell lung carcinoma show that ELF1 expression is positively correlated with histological grading and clinical outcome, indicating oncogenic function [22–25]. ELF1 has also been shown to be required for the proliferation of cervical cancer cells infected with the HPV virus [26, 27]. In contrast to these oncogenic roles, ELF1 nuclear expression is negatively correlated with histological grading and tumor size in breast ductal carcinomas [28]. In various epithelial tumors ELF1, and related ETS factors ELF2 and ELF4, inhibit proliferation and undergo mutually exclusive mutations or deletions [29]. ELF1 has also been implicated as a potential tumor suppressor in prostate cancer through analyses of mRNA and DNA copy number alterations from patient samples [30].

Recent findings indicate that metastatic prostate tumors develop resistance to therapies by inactivating the tumor suppressor genes *TP53* and *RB1* [31, 32]. In patient tumors, inactivation most commonly occurs through point mutation of *TP53* and genomic deletion of *RB1* [33, 34]. Interestingly, the *ELF1* gene is located 8 mb from *RB1* on chromosome 13 and can be co-deleted in metastatic prostate tumors. However, the contribution of ELF1 deletion to prostate cancer has not been investigated.

This study investigates the role of ELF1 in prostate cancer, providing the first analysis of ELF1*'*s function within the prostate. *ELF1* was the most commonly down-regulated ETS factor in prostate tumors and even displayed significant down-regulation in tumors lacking genomic deletions. Using a variety of phenotypic assays and next generation sequencing experiments we determined that ELF1 represses cell migration through target genes with ETS/AP-1 cis-regulatory sequences, consistent with a model of binding site competition with oncogenic ETS factors. While ELF1 functioned as a repressor relative to the oncogenic ETS factors at cell migration genes, it bound to a unique set of cis-regulatory sequences where it functioned as an activator of genes promoting senescence. Furthermore, knockdown of ELF1 increased resistance to docetaxel, a common therapeutic for late-stage prostate cancer. These data indicate that decreased *ELF1* levels in prostate tumors provide a mechanism to evade chemotherapy-induced cellular senescence or cell death, allowing for cancer recurrence.

## RESULTS

### ELF1 is negatively correlated with prostate cancer progression

**Figure 1:**
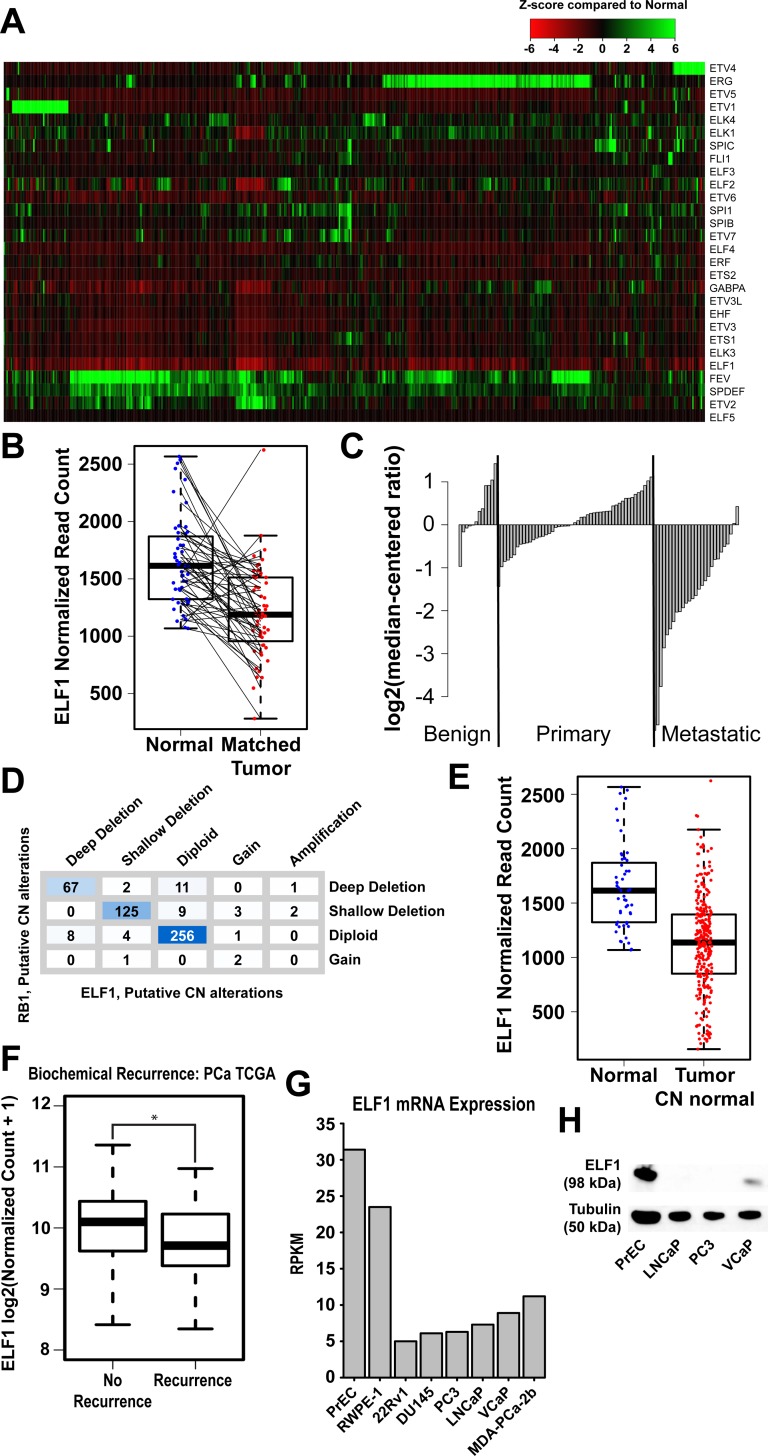
ELF1 is the most commonly downregulated ETS factor in prostate cancer **A.** Heatmap representation of mRNA expression of the ETS family in 498 prostate cancer samples relative to the background distribution of expression from 52 normal prostate samples. The values displayed represent the individual Z-score for each normalized read count as compared to the normal prostate background distribution. **B.** Boxplot representation of ELF1 normalized read counts in 52 normal prostate samples (Blue) paired with matched tumor samples (Red) (*p*-value >0.001, paired t-test). **C.** Column graph of log2 (median-centered ratio) expression of ELF1 in a microarray experiment comparing normal prostate samples with benign, primary, and metastatic prostate samples [39]. **D.** Table detailing the copy number alterations in *ELF1* and *RB1* from the TCGA data set using GISTIC 2.0. **E.** Boxplot representation of ELF1 normalized read counts in 52 normal prostate samples (Blue) compared with ELF1 expression of all prostate cancer samples that have a normal ELF1 copy number (Red) (*p*-value < 0.001, Welch Two Sample t-test). **F.** A boxplot representation of ELF1 levels in TCGA prostate tumors that are classified based on biochemical recurrence. *P*-values (* <0.05, ** < 0.01, *** < 0.001) are calculated with the Welch Two Sample t-test. **G.** RPKM expression values for ELF1 in the indicated prostate cell lines were obtained from Prensner et al. [38]. **H.** Immunoblot with the indicated antibodies (left) in a subset of the prostate cancer cell lines. Tubulin serves as a loading control.

In prostate cancer, three members of the ETS family (*ERG*, *ETV1*, and *ETV4*) are commonly overexpressed due to chromosomal translocations. A fourth member, ETV5 is over-expressed in rare cases, and has been suggested to also be an oncogenic family member [15, 20]. In contrast, some normally expressed ETS family members are reported to be down-regulated in prostate tumors. To better understand the changes that might be occurring within the ETS family during prostate tumorigenesis, we compared the mRNA expression of 498 prostate tumors against 52 normal adjacent samples using the TCGA Prostate Adenocarcinoma dataset (Figure 1A) [http://cancergenome.nih.gov/.]. As expected, the oncogenic ETS factors were overexpressed in a mutually exclusive pattern in approximately 50% of prostate tumors. One unexpected finding from this data was the widespread decrease in mRNA levels for *ELF1* in tumors (Figure 1A). A direct comparison of 52 prostate tumors with their matched adjacent normal showed that 43 out of 52 samples have decreased *ELF1* levels (Figure 1B). *ELF1* is the fourth highest expressed ETS mRNA in normal prostate cells and the decreased expression in prostate tumors is among the most significant in the ETS family (Supplemental Figure 1). Analysis of *ELF1* expression from a separate dataset, which included castrate resistant metastatic samples, showed that *ELF1* levels are decreased in primary tumors, and are dramatically decreased in metastatic samples (Figure 1C) [39]. These results suggest that *ELF1* expression is negatively correlated with prostate cancer progression.

To better understand the possible cause of the drastic mRNA decrease, copy number alterations and mutations were analyzed within the TCGA dataset. *ELF1* possesses relatively few mutations within this prostate cancer dataset (0.2% samples with ELF1 mutation); however, prostate tumors have a high rate of *ELF1* deletions (~15% deep deletion and ~27% shallow deletion). We noted that the *ELF1* gene is located 8 mb from *RB1*, which is known to be deleted in some late stage prostate tumors [31, 33, 34, 39], and from this dataset we see that *ELF1* is often co-deleted with *RB1* (Figure 1D). To test whether loss of ELF1 simply represents a passenger effect of *RB1* deletion we analyzed ELF1 expression from tumors with two copies of the *ELF1* gene. These normal *ELF1* copy number tumors still have significantly lower *ELF1* expression (*p*-val <0.0001, Welch Two Sample T-Test) than adjacent normal tissue (Figure 1E). This result predicts additional mechanisms outside of genomic deletions which result in lower *ELF1* levels, indicating selective pressure for this loss during tumor progression. Furthermore, prostate cancer patients with recurrent tumors have decreased *ELF1* levels, indicating that ELF1 loss could contribute to prostate cancer progression (Figure 1F).

To confirm that prostate cell line models recapitulate a similar difference in ELF1 expression between normal prostate and prostate cancer, mRNA and protein expression of ELF1 were compared across prostate cell lines. Analysis of previously published RNA-seq data [38] indicates that *ELF1* mRNA levels are highest in normal prostate epithelial cells (PrEC) and immortalized-normal prostate epithelial cells (RWPE-1) as compared to six prostate cancer cell lines (Figure 1G). Further, immunoblots indicate that PrEC cells have much higher ELF1 protein levels than prostate cancer cell lines (Figure 1H).

### ELF1 is a negative regulator of migration and clonogenic survival in the presence of oncogenic ETS factors

**Figure 2:**
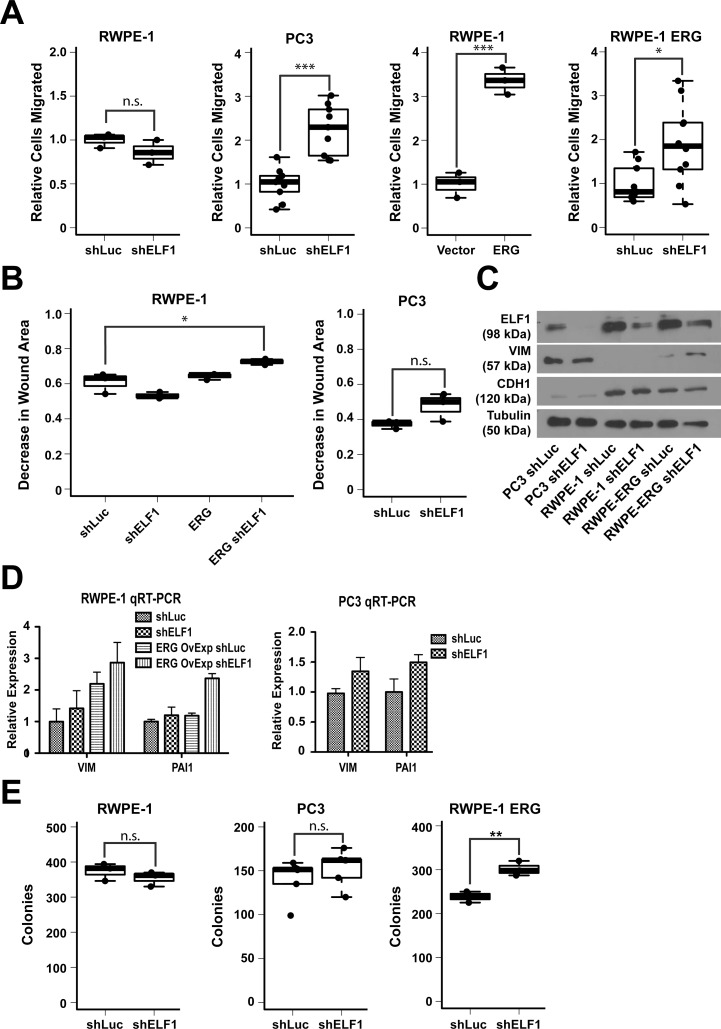
ELF1 represses oncogenic ETS mediated phenotypes in prostate cells **A.** Boxplot representation of transwell migration of RWPE-1, PC3, and RWPE-ERG cells expressing a shRNA targeting ELF1 (shELF1), or negative control shRNA targeting luciferase (shLuc) and/or expressing exogenous ERG or empty vector control. **B.** Boxplot representation of scratch assays measured as a decrease in the wound area in RWPE-1 and PC3 cells with a shRNA control (shLuc) or ELF1 shRNA knockdown (shELF1), with or without ERG. **C.** Immunoblots with the indicated antibodies. Tubulin is a loading control. **D.** Expression of indicated mRNA in the indicated cell lines relative to cells expressing shLuc alone. Values shown as mean and SEM (*n* = 3). **E.** Boxplot representation of clonogenic survival assays of cells expressing shELF1 or shLuc in RWPE-1, PC3, and RWPE-ERG cells measured as the number of colonies. All *P*-values (* <0.05, ** < 0.01, *** < 0.001) were calculated with the Welch Two Sample t-test.

ETS factors can regulate a wide-variety of cancer related phenotypes within prostate epithelial cells, but cell migration has been extensively used as a proxy to determine oncogenic or tumor suppressive activity [15, 20, 36]. To simulate the decreased expression of *ELF1* within tumors, cell migration assays with ELF1 knockdowns were performed in two different prostate cell lines; the immortalized, normal prostate epithelial cell line, RWPE-1, and the metastatic prostate cancer cell line, PC3 (Figure 2A). While there was no significant change in cell migration within the RWPE-1 cell line, there was a significant increase in cell migration in PC3 cells upon ELF1 knockdown. One major difference between these two cell lines is the overexpression of an oncogenic ETS factor, *ETV4*, within PC3 cells [7]. To determine if the presence of an oncogenic ETS factor affects ELF1's ability to suppress cell migration, ERG was expressed in RWPE-1 cells and migration assays were repeated (Figure 2A). ERG expression increased RWPE-1 cell migration (Figure 2A, third panel) and in this cellular background ELF1 repressed cell migration (Figure 2A, fourth panel). To confirm these results, scratch assays were performed under the same conditions and the trends matched the transwell assays (Figure 2B). These data indicate that ELF1 has the ability to repress prostate cell migration in the presence of oncogenic ETS factors.

Epithelial to mesenchymal transition (EMT) is characterized by epithelial cells losing polarity and becoming more migratory. To test ELF1 regulation of EMT, protein levels of an epithelial marker, CDH1 (E-cadherin), and a mesenchymal marker, VIM (vimentin), were monitored in each condition (Figure 2C). ELF1 knockdown did not alter CDH1 levels, but VIM increased upon ELF1 knockdown in RWPE-1 cells overexpressing ERG. Steady state mRNA levels of mesenchymal markers *VIM* and *PAI1* were determined by qRT-PCR in RWPE-1, RWPE-ERG, and PC3 cells, with and without ELF1 knockdown (Figure 2D). These mesenchymal genes were expressed at higher levels upon ELF1 knockdown in cells expressing oncogenic ETS, indicating that ELF1 can repress the ability of oncogenic ETS to promote EMT.

Oncogenic ETS factors can increase the clonogenic survival capacity of prostate cells along with their migratory activity [20]. To determine if ELF1 has any effect on this phenotype we conducted clonogenic survival assays under the same conditions as the migration assays. Similar to the migration assays, ELF1 was able to repress clonogenic survival of RWPE-1 cells only in the presence of ERG (Figure 2E). These results suggest that ELF1 can inhibit multiple functions of oncogenic ETS factors.

### ELF1 can bind the same ETS/AP-1 cis-regulatory elements as oncogenic ETS factors

**Figure 3:**
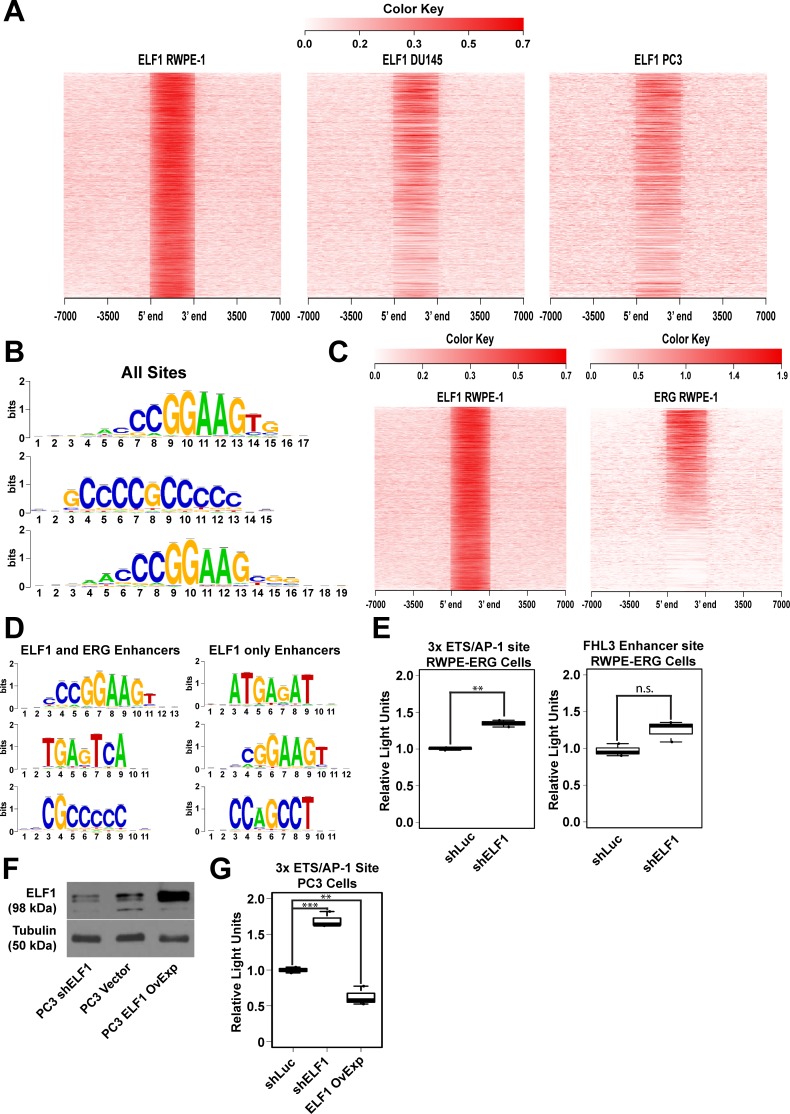
ELF1 can bind to two distinct sets of cis-regulatory sequences **A.** Heatmap representation of the ChIP enrichment of ELF1 in RWPE-1, DU145, and PC3 cells, centered on all called ELF1 bound regions in RWPE-1 cells. Bound regions are indicated between the “5′ end” and “3′ end” indicators with an extended view that includes the surrounding 7 kb on either side of the bound region. The heatmaps were generated using NGSplot [50]. **B.** Three most enriched motifs at ELF1 binding sites in RWPE-1 cells as determined by RSAT peak-motifs algorithms **C.** Heatmap representation of the ChIP enrichment of ELF1 in RWPE-1 cells and ERG in RWPE-ERG cells, centered on all called ELF1 bound regions. Bound regions are indicated between the “5′ end” and “3′ end” indicators with an extended view that includes 7 kb on either side of the bound region. Heatmaps were generated by NGSplot [50]. **D.** Three most enriched motifs at ELF1 bound enhancers (>500 bp from TSS) in two different categories, ELF1 enhancers that are bound by ERG and ELF1, and enhancers with only background ERG signal (ELF1 only). **E.** Relative luciferase reporter activity for two firefly luciferase reporter constructs (synthetic 3xETS/AP-1 sites and a fragment of a FHL3 enhancer) in RWPE-ERG cells with ELF1 shRNA knockdowns **F.** Immunoblot with the indicated antibodies (left) in the same cell lines as (G). Tubulin serves as a loading control. **G.** Relative luciferase reporter activity for the 3xETS/AP-1 firefly luciferase reporter constructs in PC3 cells with ELF1 shRNA or overexpression as indicated. Luciferase values are the ratio of firefly luciferase to minimal promoter controlled renilla luciferase signal, and this ratio was then normalized to shLuc control.

Given that ELF1 only repressed migration and clonogenic survival in the presence of an oncogenic ETS factor, it is possible that ELF1 competes for binding with oncogenic ETS factors and attenuates transcription due to weaker transactivation function. To test this hypothesis, chromatin immunoprecipitation coupled with next-generation sequencing (ChIP-seq) was used to map ELF1 genomic binding in RWPE-1 and PC3 cells and compared to a published ChIP-seq of ELF1 in DU145 prostate cancer cells [36]. ELF1 occupancy was similar between the cell lines (Figure 3A), however, the signal was highest in RWPE-1 cells, corresponding to the higher level of *ELF1* in this cell line (Figure 1G). For this reason, the RWPE-1 cell line was selected for further analysis.

Using a *p*-value cut-off of 1×10^−5^, 1837 binding sites were called for ELF1 in RWPE-1 cells using the MACS peak-caller. As expected, motif enrichment analysis for these called binding sites identified CCGGAAGT as the most overrepresented motif, matching the known DNA sequence preference of ELF1 and most of the ETS family (Figure 3B) [6]. Previous analyses of ETS factor binding patterns have identified high affinity ETS binding sequences in the promoters of many housekeeping genes that can non-specifically bind any ETS factor, while the binding sites associated with tissue specific functions of individual ETS factors occur mostly in enhancers [40, 41]. We observed a similar pattern for ELF1 (Table 1), therefore we focused our analysis on ELF1 enhancer sites (>500 bp from TSS).

**Table 1:** ELF1 binds housekeeping gene promoters and tissue specific enhancers

Most overrepresented pathways (Promoters)	*p* value
Gene Expression	8.7×10^−25^
rRNA Processing	6.7×10^−7^
Nonsense-Mediated Decay (NMD)	6.7×10^−7^
rRNA Processing in the Nucleus and Cytosol	6.7×10^−7^
Processing of Capped Intron-Containing-Pre-mRNA	3.1×10^−6^
Ribosome – Homo sapiens (human)	3.9×10^−6^
SRP-Dependent Cotranslational Protein Targeting To Membrane	4.3×10^−6^
Cytoplamsic Ribosomal Proteins	6.9×10^−6^
**Most overrepresented pathways (Enhancers)**	***p* value**
IL-7	3.7×10^−5^
Notch Signaling Pathway	0.00020
Ras Signaling Pathway – Homo sapiens (human)	0.00020
Fc-epsilon Receptor I Signaling in Mast Cells	0.00023
G1 to S Cell Cycle Control	0.00052
IL5	0.00055
IL2 Signaling Events Mediated by STAT5	0.00057
Adherens Junctions Interactions	0.00071

To test if ELF1 binds the same enhancers as ERG, consistent with competition, we compared ELF1 binding to our previously published ChIP-seq of ERG in RWPE-ERG cells [20]. A heatmap representation of the signal for each factor at called ELF1 sites demonstrated overlapping binding with ERG at about one-half of ELF1 targets, but also showed a subset of ELF1 sites with no evidence of ERG binding (Figure 3C). Using a 2-fold cutoff for the ratio of binding site signal to adjacent background signal for ERG, we determined that ELF1 has 853 binding sites that overlap with ERG (443 promoter and 410 enhancer) and 984 unique binding sites with limited or no ERG signal (135 promoter and 849 enhancer). Motif enrichment analysis of the enhancer regions for each category showed a unique set of binding motifs. In the overlapping enhancer binding regions there was an enrichment for ETS, AP-1, and SP1 binding sequences, while the unique ELF1 bound enhancers were enriched for GATA, ETS, and TFAP2C sequences (Figure 3D). Neighboring ETS and AP-1 sites are enriched at cis-regulatory regions controlling genes associated with cell migration [36]. Pathway analysis of the genes nearest to ELF1/ERG overlapping sites did indeed show enrichment for migration related pathways, such as cell to cell communication, cell junction organization, and various growth factor and cytokine signaling pathways (Table 2).

**Table 2:** ELF/ERG overlapping and ELF1 unique enhancers are near genes with distinct biological functions

Most overrepresented pathways (ELF/ERG overlapping Enhancers)	*p* value
IL-5 Signaling Pathway	0.00047
Cell Junction Organization	0.00060
Signaling by FGFR1	0.00133
Cell-Cell Communication	0.00136
Leptin Signaling Pathway	0.00154
VEGFA-VEGFR2 Signaling Pathway	0.00182
**Most overrepresented pathways (ELF1 Unique Enhancers)**	***p* value**
Signaling by NOTCH	0.00051
G1 to S Cell Cycle Control	0.00076
Adherens Junctions Interactions	0.00077
Breast Cancer – Homo sapiens (human)	0.00089
Melanoma – Homo sapiens (human)	0.00105
Cyclin D Associated Events in G1	0.00266

Combined with the migration assay results, these data indicated that ELF1 and ERG may compete for binding at ETS/AP-1 sites, with ERG acting as a transcriptional activator and ELF1 acting as a weaker activator or a repressor. To confirm ELF1's repressive activity at ETS/AP-1 sites in cells expressing ERG, a luciferase assay was performed with two ETS/AP-1 regulated reporter constructs (Figure 3E). The first was an artificial construct with three copies of the ETS/AP-1 sequence upstream of a minimal reporter. The second has the ETS/AP-1 containing enhancer of the FHL3 gene, which we have previously used to test transcriptional activation by ERG [20, 35]. Knockdown of ELF1 in RWPE-ERG cells increased expression of both reporters. In PC3 cells, which overexpress ETV4, ELF1 knockdown and overexpression cell lines were generated (Figure 3F). Similar to the RWPE-ERG cells lines, ELF1 knockdown increased the 3x ETS/AP-1 reporter, while ELF1 over-expression decreased the reporter (Figure 3G). Therefore, ELF1 can repress ETS/AP-1 regulated reporters in oncogenic ETS-overexpressing cell lines.

### ELF1 represses EMT genes, but activates genes promoting cellular senescence

**Figure 4:**
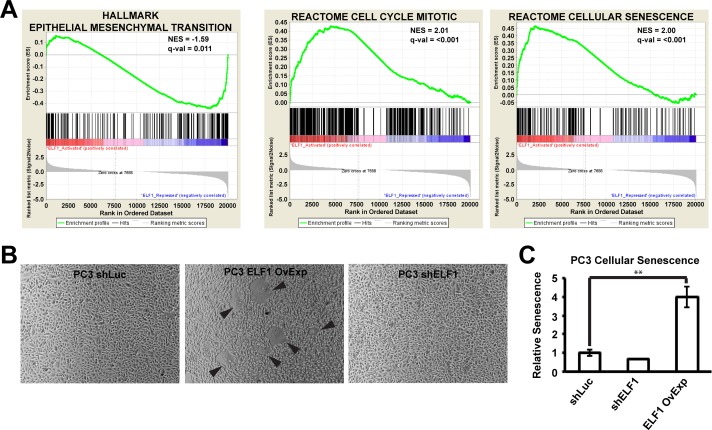
ELF1 represses EMT, but activates cellular senescence **A.** Gene Set Enrichment Analysis (GSEA) was used to compare RNA-seq of RWPE-ERG and RWPE-ERG shELF1 for the indicated gene sets from MSigDB Hallmarks or Reactome. The fpkm values were provided for the three biological replicates in each condition and the Signal2Noise ratio was used to rank genes. **B.** Immunoblot with the displayed antibodies (left) in PC3 cells with a control shRNA (shLuc) or ELF1 shRNA knockdown (shELF1), or a retroviral overexpression of ELF1 (ELF1 OvExp). **C.** Microscopic images PC3 cells from (B). Images were converted to grayscale and the saturation was adjusted to provide a clearer outline of the cells. The black arrows indicate cells that appear to be undergoing senescence. **D.** Quantification of β-galactosidase positive staining in PC3 cells expressing indicated constructs relative to shLuc (*n* = 3). *P*-values determined by one-way ANOVA with Tukey's Test as the post-hoc analysis (* <0.05, ** < 0.01, *** <0.001).

To better understand ELF1's function in prostate epithelial cells we performed differential expression analysis using RNA sequencing of RWPE-1 cells, RWPE-ERG cells, and RWPE-ERG cells with ELF1 knockdown. 1880 genes were significantly downregulated and 2096 genes were significantly upregulated upon knockdown of ELF1 in RWPE-ERG cells. Gene set enrichment analysis was performed on the changes in gene expression when ELF1 was depleted from RWPE-ERG cells. EMT hallmark genes were enriched within the ELF1 repressed category (Figure 4A). Furthermore, ontology analysis of the genes significantly activated by ERG and repressed by ELF1 showed enrichment for genes implicated in cell morphogenesis, mesenchyme development, and VEGF signaling (Table 3). These results matched the migration assays and qRT-PCR analysis from the RWPE-1 and PC3 cell lines (Figure 2), where the oncogenic ETS drive EMT and migration while ELF1 opposes this function.

**Table 3:** ELF1 represses genes associated with EMT and activates genes associated with cell cycle and senescence

Most overrepresented categories (Sig. ERG Act. & Sig ELF1 Rep.)	Adj. *p*-value
Glycosaminoglycan biosynthesis - chondroitin sulfate / dermatan sulfate	0.0155
Elastic fibre formation	0.0197
Mesenchyme development	0.0337
Plasminogen activation	0.0337
Vasculature development	0.0337
Neurogenesis	0.0337
Circulatory system development	0.0337
Stem cell development	0.0337
Modulation of excitatory postsynaptic potential	0.0337
Cell morphogenesis involved in neuron differentiation	0.0337
Anatomical structure formation involved in morphogenesis	0.0337
Axonogenesis	0.0337
Blood vessel development	0.0337
Positive regulation of excitatory postsynaptic potential	0.0337
Vascular endothelial growth factor receptor signaling pathway	0.0337
**Most overrepresented categories (top 1000 Sig. ELF1 Activated)**	**Adj. *p*-value**
Meiotic recombination	0.0000767
SIRT1 negatively regulates rRNA Expression	0.0000767
DNA methylation	0.0000767
RNA Polymerase I Promoter Opening	0.0000767
DNA Damage/Telomere Stress Induced Senescence	0.0000767
PRC2 methylates histones and DNA	0.0000802
B-WICH complex positively regulates rRNA expression	0.0000849
Packaging Of Telomere Ends	0.0000849
Activated PKN1 stimulates transcription of AR (androgen receptor) regulated genes KLK2 and KLK3	0.0000849
Cellular Senescence	0.000179
Oxidative Stress Induced Senescence	0.000188
RNA Polymerase I, RNA Polymerase III, and Mitochondrial Transcription	0.000227
Condensation of Prophase Chromosomes	0.000227
Senescence-Associated Secretory Phenotype (SASP)	0.000227
Amyloid fiber formation	0.000258

Looking in the opposite direction, ELF1 activated gene sets were enriched for categories related to cell cycle control, and cellular senescence (Figure 4A). ELF1 has previously been demonstrated to regulate cell proliferation in epithelial cells and thus identification of cell cycle pathways was expected [26, 29]; however, the abundance of senescence related pathways was an unexpected finding. Ontology analysis of the top 1000 significantly activated genes by ELF1 from the RNA-seq also displayed a number of senescence related pathways (Table 3). Interestingly, when ELF1 was overexpressed in PC3 cells (Figure 3F), a number of cells took on a large, flattened, fried egg-like morphology, consistent with cells undergoing senescence (Figure 4B). To test if these cells were undergoing senescence, we tested for β-galactosidase activity, a common marker of senescence. There was a significant increase in β-galactosidase staining of PC3 cells when ELF1 was overexpressed, and a slight decrease upon knockdown, demonstrating that over-expression of ELF1 activated senescence (Figure 4C).

### ELF1 loss results in increased resistance of prostate cancer cells to treatment

Gene set enrichment analysis of the TCGA prostate cancer dataset, ranked based on the Pearson correlation of each gene relative to ELF1 mRNA levels, provided further support that ELF1 can function as a tumor suppressor within the prostate (Table 4). Genes which positively correlated with ELF1 expression in prostate tumors were enriched for gene sets downregulated in prostate cancer, metastasis related pathways, and chemotherapy resistance; while genes that negatively correlated with ELF1 showed enrichment for gene sets upregulated in these same categories. While the prostate cancer and metastasis related gene sets were expected based on our previous genomic and phenotypic assay findings, the ability of ELF1 to potentially affect chemotherapy resistance was novel. It has been recently reported that *RB1* deletion can promote resistance to chemotherapy in prostate cancer [31, 34]; however, as the *ELF1* locus is often lost in these same genomic deletions, we asked if ELF1 depletion might contribute to chemotherapy resistance.

**Table 4:** ELF1 expression in prostate tumors negatively correlates with prostate cancer and chemotherapy resistancegenes

ELF1 Positively Correlated Gene Sets	Size	NES	FDR q-val
LIU Prostate Cancer DN	466	2.91	<0.0001
KANG Doxorubicin Resistance DN	19	2.26	<0.0001
TOMLINS Prostate Cancer DN	40	2.22	<0.0001
ALONSO Metastasis DN	26	1.80	0.0034
WANG Tumor Invasiveness DN	208	1.60	0.0208
**ELF1 Negatively Correlated Gene Sets**	**Size**	**NES**	**FDR q-val**
HONMA Docetaxel Resistance	34	−2.24	<0.0001
LIU Prostate Cancer UP	90	−2.22	<0.0001
RHODES Cancer Meta Signature	64	−1.90	0.0011
WANG Tumor Invasiveness UP	370	−1.79	0.0026
TOMLINS Prostate Cancer UP	40	−1.76	0.0035

**Figure 5:**
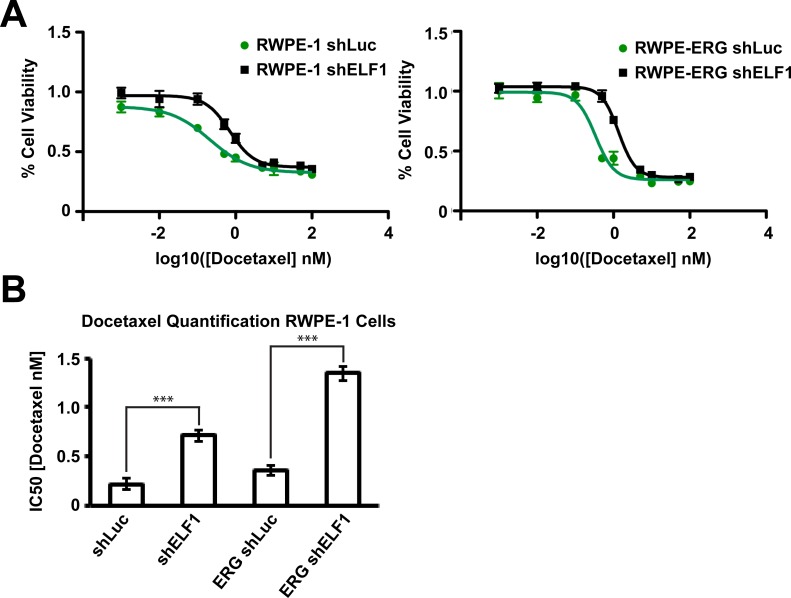
ELF1 sensitizes prostate cells to chemotherapy **A.** Cell viability analysis of RWPE-1 or RWPE-ERG cells with a lentiviral luciferase knockdown (shLuc) or ELF1 knockdown (shELF1) (*n* = 3). **B.** Barplot representation of the Mean and SEM IC50 value for analysis in (A). IC50 values were calculated using a nonlinear fit of the log(inhibitor) vs. response - Variable slope (four parameters) in Prism. *P*-values were determined using a one-way ANOVA with Tukey's Test as the post-hoc analysis (* <0.05, ** < 0.01, *** <0.001).

To test if ELF1 loss might contribute to chemotherapy resistance, RWPE-1 cells, with or without ERG expression, were treated with docetaxel. We found that depletion of ELF1 in both conditions resulted in significantly increased IC50 values, indicative of a decreased sensitivity to docetaxel (Figure 5A). ELF1 depletion increased the IC50 by 3.2-fold in RWPE-1 cells, and 3.8-fold in RWPE-ERG cells (Figure 5B). These results further support the idea that ELF1 loss could result in increased resistance to prostate cancer treatments and correlate with the finding that *ELF1* levels are lower in patients with recurrent tumors (Figure 1F).

## DISCUSSION

Here we report that ELF1 can have tumor suppressive functions in prostate epithelial cells and that ELF1 levels are negatively correlated with cancer progression within this tissue. ELF1 binds to some of the same ETS binding sites as ERG, where ELF1 functions as a transcriptional repressor of cell migration genes; however, at sites where it binds uniquely, ELF1 activates and upregulates genes involved in senescence. Therapeutic strategies for invasive prostate cancer commonly include docetaxel treatment, which result in senescence or death of the cancer cells [42, 43]. ELF1 levels may be reduced in tumors to allow for the bypass of these pathways upon exposure to chemotherapies. This function, along with ELF1's ability to repress cell migration at ETS/AP-1 sites, provides a potential mechanism for tumor suppressive functions of ELF1.

Within prostate cancer, it has been well established that the oncogenic ETS factors, most often ERG, become overexpressed through chromosomal rearrangements and elicit several phenotypic changes that allow for the progression from a benign neoplasia to an invasive carcinoma, when paired with PTEN deletion [39, 44, 45]. However, the role of the endogenously expressed ETS factors within the prostate and their ability to compete with the oncogenic ETS factors has been much less studied. Several ETS family members have been shown to act as tumor suppressors within the prostate (EHF, SPDEF, ERF, etc) [8, 10, 12, 17, 46]. Of these, EHF and ERF have been shown to directly compete with oncogenic ETS at cis-regulatory sequences, opposing the ability of the oncogenic ETS to activate nearby genes [16, 17]. Here we find that the commonly downregulated ETS factor ELF1 can bind and repress the same ETS/AP-1 sequence elements that are activated by oncogenic ETS factors to induce EMT and cell migration. The downregulation of ELF1 in ETS fusion positive prostate tumors could allow for increased binding of oncogenic ETS factors at these sites, leading to a subsequent activation of genes involved in various cancerous phenotypes.

Previous cancer studies indicate that *ELF1* can be a tumor suppressor or an oncogene, depending on the cell type [22–24, 26, 28]. The proposed oncogenic function of ELF1 stems from its ability to promote the cell cycle in a similar manner to E2F transcription factors. Like E2F factors, ELF1 binds hypophosphorylated RB1, and this represses ELF1 mediated transcription of cell cycle genes. However, hyperphosphorylation of RB1 by cyclin dependent kinases leads to dissociation from ELF1 and allows ELF1 to activate transcription as cells pass through the cell cycle [47]. Therefore, *RB1* status is a key to ELF1 function. This is evidenced in cervical cancer, where the presence of the human papilloma virus E7 protein inactivates RB1 and allows ELF1 to switch from a repressor of proliferation to an activator of proliferation [26]. Recent findings indicate that metastatic prostate tumors develop resistance to therapies by inactivating *TP53* through point mutations and *RB1* through genomic deletion [31, 33, 34]. *RB1* in other tumor types is highly mutated to produce nonfunctional protein products or to disrupt the E2F binding pocket; however, the aforementioned genomic deletions of *RB1* are most prevalent within prostate cancer [39]. Interestingly, the *ELF1* locus is roughly 8 Mb away from *RB1* on chromosome 13 and is often co-deleted in prostate tumors (Figure 1D). Our findings that ELF1 promotes senescence in prostate cells could explain why *RB1* is lost due to deletion, rather than mutation in prostate tumors. We hypothesize that ELF1 could act as a sensor of *RB1* status in prostate cells. Mutational inactivation of RB1 would result in high transcriptional activity of ELF1 which would activate cellular senescence. However, the large chromosomal deletions found in prostate tumors delete both *RB1* and *ELF1*, and this response would be lost.

Further evidence of ELF1 having the ability to regulate pathways involved in cell fate and cell cycle progression comes from an unbiased search for factors that are phosphorylated and experience changes in mRNA expression upon ionizing radiation in human embryonic kidney 293T cells and osteoscarcoma U2OS cells. ELF1 was identified as a potential downstream target of the DNA damage response pathway, and following ionizing radiation U2OS cells with a siRNA against ELF1 were more likely to escape cell cycle arrest by bypassing the G2-M checkpoint [48]. Similarly, ELF4, a member of the same ETS subfamily as ELF1, was demonstrated to be a regulator of the DNA damage response and is used to recruit ATM to sites of DNA damage. In this context, persistent ELF4 blocked the repair of damaged DNA which led to increased apoptosis [49]. Thus the loss of ELF4 allowed for increased DNA damage repair and increased resistance to treatment, similar to ELF1 in this study.

Our findings, along with previous studies of ELF1 and its subfamily, suggest that ELF1 has the ability to compete with other expressed ETS factors and is also able to regulate cell fate decisions in prostate cancer cells. The loss of ELF1 through chromosomal deletion or mRNA downregulation can contribute to prostate cancer progression by allowing for increased binding of stronger ETS transactivators at cell migration genes and by decreasing ELF1's ability to activate cellular senescence or death upon treatment with chemotherapies.

## MATERIALS AND METHODS

### Cell culture and viral transduction

The PC3 and RWPE-1 cell lines were purchased from the American Type Culture Collection (ATCC) within a year of starting this study. The cell lines were frozen within 4 passages of obtaining them from ATCC to allow for consistent results. These cells were authenticated by viral testing (RWPE-1 cells), isoenzymes (RWPE-1 cells), DNA profile, and cytogenetic analysis by the ATCC before purchase. Cell lines were cultured by ATCC recommendation as follows: EBNA293 and HEK-293T were grown in Dulbecco's modification Eagle (Sigma) with 10% fetal bovine serum (Sigma). PC3 cells were grown in F12K (Sigma) with 10% FBS. RWPE-1 cells were grown in Keratinocyte media (ThermoFisher). All media included 1X Penicillin/Streptomycin (Mediatech-Cellgro).

The lentiviruses for the luciferase and ELF1 shRNAs were produced by co-transfection of pLKO.1 (Addgene plasmid 8453) with the shRNA sequences as follows;

Luciferase Forward Primer:CCGGCTTACGCTG AGTACTTCGATTCAAGAGATCGAAGTACTCAGC GTAAGTTTTTTTG, Luciferase Reverse Primer:AATT CAAAAAAACTTACGCTGAGTACTTCGATCTCTT GAAGTACTCAGCGTAAG, ELF1 Forward Primer:CC GGAAACAGTGCCACTCACAACAGCTCGAGCTG TTGTGAGTGGCACTGTTTTTTTTG, ELF1 Reverse Primer:AATTTCAAAAAAAACAGTGCCACTCACA ACAGCTCGAGCTGTTGTGAGTGAGTGGCACTG TTT, in HEK293T cells with pMDLg/pRRE (Addgene plasmid 12251), pRSV-Rev (Addgene plasmid 12253) and pMD2.G (Addgene plasmid 12259) packaging plasmids as previously described [35]. Retroviral overexpression vectors for ELF1 and ERG were produced using the method previously described in [20].

### RNA sequencing and analysis

Total RNA for three independent biological replicates was isolated from RWPE-1 cells transduced with lentiviral shRNA knockdown or retroviral overexpression vectors (see above) using the RNeasy mini kit (Qiagen) according to manufacturer's instructions. RNA was DNase treated with the RNAse-Free DNase kit (Qiagen) per the manufacturer's protocol. The RNA was polyA selected using purified oligo(dT) beads (Invitrogen). Sequencing libraries were generated using the Illumina TruSeq sample preparation protocol. The sequencing reads were analyzed with the Tuxedo Suite RNA sequencing pipeline to obtain differential gene expression. Data files are available for download from NCBI's Gene Expression Omnibus (GEO; http://www.ncbi.nlm.nih.gov/geo/), accession number GSE113499.

### Protein immunoblotting and RNA quantification

Total protein extract from equal number of cells was separated on 10% SDS-PAGE gels, transferred to nitrocellulose membrane (Bio-Rad), blocked in 5% milk in TBS (10mM Tris, pH8.0, 150 mM NaCl), incubated with primary and secondary antibodies, and visualized by ECL (Thermo Scientific) using standard procedures. Antibodies used in this study were ELF1 (sc-631, Santa Cruz Biotechnology), ERG (CM 421, Biocare), CDH1 (610181, BD Transduction Laboratories), VIM (clone V9, M0725, Dako), and Tubulin (T9026, Sigma).

RNA levels were measured by reverse transcription followed by quantitative PCR with standard curves as described previously [15], using the following DNA oligonucleotides; 18S Forward Primer: GGTGAAATTCTTGGACCGGC, 18S Reverse Primer: GACTTTGGTTTCCCGGAAGC, VIM Forward Primer: CGCCATCAACACCGAGTTC, VIM Reverse Primer: ATCTTATTCTGCTGCTCCAGGAA, PAI1 Forward Primer: CCTAGAGAACCTGGGAATGACC, PAI1 Reverse Primer: CCTCGATCTTCACTTTCTGCAGC. RNA levels were normalized to 18S rRNA.

### Luciferase reporter assay

A 474bp fragment of an FHL3 enhancer (chr1:38465034-38465507, hg19) and a synthetic 3xETS/AP-1 region (core binding sequence GGAAGTGACTCA) were cloned into the firefly luciferase reporter pGL4.25 (Promega). The methods for generating these plasmids are described in [20] for the FHL3 enhancer and [35] for the 3xETS/AP-1 plasmid. The dual luciferase reporter assay (Promega) measures luciferase activity as described [35].

### Transwell migration assays

Transwell migration assays were carried out as previously described [15], with minor modifications. In brief, 5×104 cells were introduced to the transwell (8 µM pore size; BD Bioscience) and incubated for 48 hrs (PC3 cells) or 64 hrs (RWPE-1 cells). Cells on the underside of the transwell were then fixed, stained, and counted. A mean count of five locations on the membrane determined the number of migrated cells per technical replicate, and the average of 2 technical replicates was used to determine the number of migrated cells for each biological replicate.

### Scratch assays

Scratch assays were performed in 6 well plates with an initial count of 1×10^6^ cells. Cells adhered for 24 hours before being scratched with a P1000 tip. Each biological replicate was the mean of three technical replicates on the same plate. After scratching, the media was removed and the plate was washed with PBS before fresh media was added. Images were taken at this time to quantify the size of the initial wound. The cells were then incubated for 48 hrs (RWPE-1) or 24 hrs (PC3). The cells were then imaged at the same location. ImageJ quantified the size of each scratch.

### Chromatin immunoprecipitation and sequencing

ChIP was previously described [15]. Briefly, cells were crosslinked using 1% v/v formaldehyde (Fisher Scientific) for 15 minutes and quenched with 2M Glycine for 5 minutes. Isolated cells were lysed and sonicated (Daigenode, Bioruptor Pico) for 3 minutes (30 sec ON/OFF). Nuclear lysate was rotated with antibody for 4 hours at 40C, washed, and DNA isolated by phenol/chloroform. Antibodies used were ERG (CM 421, Biocare), and ELF1 (sc-631, Santa Cruz Biotechnology). Library preparation was carried out as previously described [36]. Peak calling was performed using Macs v1.4.2 and nearest neighboring genes were determined using the USeq platform (http://useq.sourceforge.net/) with the hg19 genome.

ChIP-seq Data files generated in this study can be found via Gene Expression Omnibus (http://www.ncbi.nlm.nih.gov/geo/) accession number GSE113499.

### Motif searching and ontology analysis

Enriched motif searching used the RSAT “peak motifs” platform (http://rsat.sb-roscoff.fr/). Settings for RSAT are as followed: Discover over-represented words and discover words with local overrepresentation at an oligomer length of 6, 7, and 8. Number of motifs returned per algorithm was set equal to 5. All other options remained as default settings. Ontology and pathway searches used ConsensusPathDB (cpdb.molgen.mpg. de) or gProfiler (https://biit.cs.ut.ee/gprofiler/). Settings for the ConsensusPathDB were default with a minimum overlap of 5 genes. gProfiler settings were default except for a functional categorize size maximum of 2000, no Hierarchical sorting, and only Biological process, KEGG, and Reactome were selected as options for searching.

### Data curation and heatmaps

RNA-sequencing data from patient tumor and adjacent normal samples were generated in whole by the TCGA Research Network (http://cancergenome.nih.gov/.). Normalized read counts for each ETS family member were used to generate the Z-score heatmap. A background distribution of normalized read counts was created from the 52 adjacent normal samples and the Z-score was generated for each tumor sample by comparing the tumor normalized read count to the adjacent normal background distribution. The heatmap was clustered using the Ward method with correlation-based distance. FPKM-UQ values for the top 10 expressed ETS factors in prostate cells were pulled from the GDC TCGA Prostate Cancer (PRAD) data set on the Xena browser [37].

RNA-sequencing data for various prostate cancer cell lines was obtained from a publicly available data set on GEO (GSE31728) [38]. Only the single-end read samples were used from this data to ensure that all of the sequencing files were analyzed consistently. The sequencing reads were analyzed using the Tuxedo Suite RNA sequencing pipeline.

### Clonogenic survival assay

Approximately 1000 cells were seeded onto 6-well tissue culture plates and incubated at 37 °C for 9-14 days. Upon completion of the incubation period, cells were fixed with 10% formalin and were stained with 0.5% crystal violet in 25% methanol. Stained colonies were counted using the Genesys image acquisition and analysis software (Syngene). The number of colonies are reported as the mean of three biological replicates, each the average of two technical replicates.

### Senescence assay: β-galactosidase staining

PC3 cells were plated into a 96-well plate three days before fixing and staining the cells as outlined per the manufacturer's protocol (Senescence β-galactosidase Staining Kit, Cell Signaling Technology #9860). Stained cells were imaged randomly in 5 locations from each well and the mean was the technical replicate. The mean of two technical replicates generated a biological replicate, and three biological replicates were used for each condition.

### Cell viability assay

Cell viability was measured using the MTT reagent (Calbiochem) and different doses of docetaxel. 5000 cells were plated onto a 96-well tissue culture plate and incubated at 37 °C for 48 hours before drug treatment. Docetaxel was added from 0.001 nM to 100 nM and cells were incubated for 72 hours at 37 °C. Media was removed from the cells and replaced with MTT reagent (5 mg/ml in PBS) for 4 hr. Absorbance was measured at 600 nm using a micro-plate reader ELx8200 (Biotek Instruments). Viability was the mean percentage of absorbance relative to an untreated well for three biological replicates.

### Gene set enrichment analysis (GSEA)

The desktop version 3.0 of GSEA was utilized along with datasets from the Molecular Signatures Database (MSigDB). A Gene Cluster Text file (.gct) was generated using the fpkm values for each of the three biological replicates in the RWPE-ERG and RWPE-ERG shELF1 conditions from the RNA-seq. The RWPE-ERG data represented the ELF1 Activated phenotype while the RWPE-ERG shELF1 data represented the ELF1 Repressed phenotype in GSEA. The default parameters were used within GSEA except that the collapse dataset to gene symbols was set to False. A ranked file (.rnk) was generated from the prostate cancer TCGA dataset by determining the Pearson correlation between each gene and ELF1. The GSEAPreranked program determined enriched gene sets with the default parameters.

### Availability of data and materials

The next generation sequencing datasets supporting the conclusions of this article are available in the NCBI's Gene Expression Omnibus (GEO) repository (http://www.ncbi.nlm.nih.gov/geo/) accession number GSE113499. The RNA-sequencing data from patient tumor and adjacent normal samples were generated in whole by the TCGA Research Network (http://cancergenome.nih.gov/.). The RNA-sequencing data for various prostate cancer cell lines was obtained from a publicly available data set on GEO (GSE31728) [38]. The microarray data for patient samples with benign prostate tissue, localized prostate cancer, or metastatic castrate resistant prostate cancer is available on the NCBI's GEO repository, accession number GSE35988 [39]. The remaining datasets supporting the conclusions of this article are included within the article.

## SUPPLEMENTARY FIGURE


