# Supplementary material for: Common ELF1 deletion in prostate cancer bolsters oncogenic ETS function, inhibits senescence and promotes docetaxel resistance
Source: Genes Cancer. 2018 May;9(5-6):198–214. doi: 10.18632/genesandcancer.182 (PMC6305106; doi:10.18632/genesandcancer.182)
Supplement: Supplementary file 1 [file ganc-09-198-s001.pdf]

Common ELF1 deletion in prostate cancer bolsters oncogenic ETS function, inhibits senescence and promotes docetaxel resistance – Budka et al

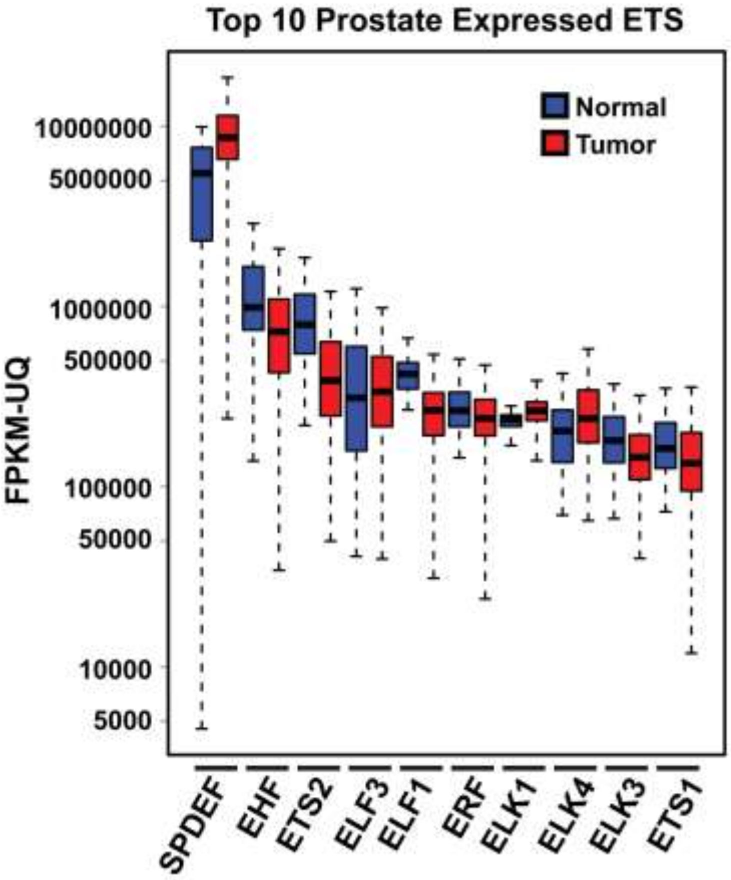

Supplemental Figure 1: Top 10 expressed ETS in normal prostate and their expression in prostate cancer. Boxplot representation of the upper quartile normalized FPKM values of the 10 most highly expressed ETS factors in normal prostate samples. The 52 normal prostate expression values (Blue) are compared to the 498 prostate cancer expression values (Red) for each of these ETS factors and the genes are ordered based on their mean expression in normal prostate samples in descending order.
